# Fluency training in medical education: Improving competence in IV fluid therapy knowledge and skills

**DOI:** 10.15694/mep.2019.000023.1

**Published:** 2019-01-29

**Authors:** Ian K Walsh, Katerina Dounavi, Joseph Houghton, Kathy M Cullen, Karola Dillenburger

**Affiliations:** 1 Queens University Belfast

**Keywords:** medical education, behaviour analysis, precision teaching, fluency, SAFMEDS

## Abstract

This article was migrated. The article was marked as recommended.

**Objectives:** Intravenous fluid (IV) therapy is an important component of care for many hospital patients, especially in perioperative and acute care settings. However, errors in fluid composition and dosing can be life-threatening. To achieve competent professional performance, i.e., accurate and fluent, it is vitally important that medical students receive effective training in IV fluid therapy.

**Methods:** In this study, we explored how Precision Teaching (PT), a behaviour analytic teaching method, can enhance outcomes of usual medical education techniques.

A total of 178 third-year medical students participated in the study during the IV fluid therapy training week. All students completed a multiple-choice test pre- and post-training. In addition to standard IV fluid therapy teaching, the experimental intervention group (n=83 students) used SAFMEDS ( *S*ay *A*ll *F*ast *M*inute *E*very *D*ay *S*huffled) cards approximately 3-5 times per day for 5 days. The other 95 students (control group) received teaching as usual, but did not undergo the additional training.

**Results:** Results show that the SAFMEDS boosted performance of the intervention group on the MCQ by 20 percentage points when compared to the control group. Fluency (accuracy and speed) of performance on SAFMED trials increased markedly during the intervention week and there was evidence that weaker students benefitted in particular.

**Conclusions:** Implications for medical education are outlined.

## Introduction

Hospital patients often require intravenous (IV) fluid therapy to avoid or address problems with fluid and/or electrolyte balance.Given that IV fluid prescriptions vary from patient to patient, deciding on the appropriate amount and composition of IV fluids and the rate at which to administer them, is a complex task fraught with potential risk ( Forryan and Mishra, 2017). TheNational Confidential Enquiry into Perioperative Deaths ( NCEPOD, 2004) found a relatively high incidence of errors in fluid prescription leading to a significant number of patients dying, e.g., from pulmonary oedema and severe biochemical disturbances, such as hyponatraemia that arise from excessive fluid administration, or from acute kidney injury that results from under resuscitation ( NICE, 2013). In fact, as many as 1 in 5 patients who receive IV fluids and electrolytes suffer complications or morbidity due to inappropriate administration ( NCEPOD, 2004). Hence, it has been argued that fluid prescribing should be attributed the same status as drug prescribing ( NICE, 2013).

Safe intravenous fluid prescribing requires the integration of relevant clinical skills, such as the assessment of fluid balance, with an understanding of fluid physiology under normal and pathological conditions, and the properties of commonly available intravenous fluids ( Frost, 2015). In an effort to improve current practice and in recognition of the importance of accurate and proficient IV fluid prescribing, the National Institute for Health and Care Excellence ( NICE, 2013)introduced of the 5Rs (Resuscitation, Routine maintenance, Replacement, Redistribution, and Reassessment) and recommended that all healthcare professionals involved in prescribing and delivering IV fluid therapy need to be fully trained and demonstrate competence in:

• ‘understanding the physiology of fluid and electrolyte balance in patients with normal physiology and during illness
• assessing patients’ fluid and electrolyte needs (the 5 Rs: Resuscitation, Routine maintenance, Replacement, Redistribution and Reassessment)
• assessing the risks, benefits and harms of IV fluids prescribing and administering IV fluids monitoring the patient response evaluating and documenting changes and taking appropriate action as required.’ (p10)

As many as 90% of IV fluid prescriptions are managed by relatively junior, foundation year 1 (FY1) doctors ( Lobo *et al.*, 2001; Walsh and Walsh, 2005; Powell *et al.*, 2013). For this reason, this knowledge forms an essential component of the curriculum towards the end of undergraduate medical training. Various pedagogic approaches have been utilised to teach IV fluid knowledge and relevant skills; for example, systems-based or problem-based learning ( McCrory *et al.*, 2017) and e-learning ( RCH Medical Education, 2014). However, research has shown that only a minority (15%) of FY1 doctors are adequately trained in this area and the approach to IV fluid prescribing amongst junior doctors across hospitals is highly variable, with poor awareness of the national guidelines ( Lobo *et al.*, 2002).

Given the shortcomings of IV fluid therapy skills in FY1 doctors and the concomitant danger to patients, the current study explored the effectiveness of adding Precision Teaching (PT) methods to the existing teaching in this field. PT has been used successfully in medical education, e.g., to teach venepuncture skills ( Lydon *et al.*, 2017), dermatological diagnostics ( McGrath *et al.*, 2018), and left-right discrimination ( Brennan *et al.*, 2018), but is a novel approach to teaching the IV fluid therapy curriculum.

Rooted in the scientific discipline of applied behaviour analysis ( Cooper, Heron and Heward, 2007), the basic tenet of Precision Teaching (PT) is to teach target skills to mastery level, i.e., to achieve behavioural fluency ( Graf and Lindsley, 2002). Behavioural fluency is attained when the target behaviour is performed with *accuracy* and at *speed.* In other words, it is not enough to achieve accurate performance of the target behaviour, but the performance also has to be accomplished at a competent pace ( Binder, 1996). In PT, behavioural fluency is achieved through timed practice of the accurate behaviour, usually thorough repeated one-minute speed trials. This amounts to *over-teaching* and has shown to be highly effective in achieving behavioural fluency ( Fabrizio, 2003).

One of the key tools used in PT are so-called SAFMEDS flashcards ( *S*ay *A*ll *F*ast *M*inute *E*very *D*ay *S*huffled) ( Beverley, Hughes and Hastings, 2009). SAFMEDS are small cards with the stimulus/probe on one side (pictures, words to define, or brief questions) and the desired response on the other side (the correct response). SAFMEDS are used in one-minute speed trials, with a timer being set and students answering as many SAFMEDS as they can in 60 seconds, putting correctly answered cards on the table in one pile and incorrectly answered cards on another pile ( Bartley, 2013). After each trial, the cards on each pile are counted and correct vs incorrect performance is recorded. Incorrectly answered cards can then be studied with 1-minute trials being continued until the target number of SAFMEDS is completed correctly within 1 minute. The target number is set before trials start, by asking competent performers, such as highly experienced doctors, to complete as many SAFMEDS as possible in 1 minute. The average of their performance is then used as a target for the students.

Using 1-minute trials means that performance is measured in a contained, highly concentrated situation without attention drift or participant tiring ( Binder, 1996). These trials can be repeated several times per day without requiring much time or interfering with other teaching methods ( Johnson and Street, 2014).

Precision Teaching (PT) has been shown to be an effective teaching method for a range of behaviours, especially academic and performance related behaviour. In school children, PT has also increased motivation and the ability to follow complex teacher instructions ( Graf and Lindsley, 2002). As such, PT lent itself perfectly for deployment in teaching IV fluid therapy.

In this study, we explored the effects of using SAFMEDS and speed trials on the performance of senior undergraduate medical students in relation to key IV fluid therapy facts and concepts, addressing the following research question:

• Does adding brief PT-based training, with the instructional tool of SAFMEDS and speed trials, improve performance when teaching final-year medical students the IV fluid therapy curriculum?

The target outcomes for the students were as follows:

• Improved knowledge of IV fluid therapy facts and concepts, assessed via pre- and post-intervention multiple choice questionnaire.
• Fluency (accuracy and speed) in the use of the bespoke IV fluid curriculum SAFMEDs assessed via numerous speed trials.

## Methods

### Ethics

Ethical approval was granted from the School of Medicine, Queen’s University Belfast. The study was conducted in adherence to QUB Research Governance Guidelines and all relevant data protection procedures were implemented. Only students who had signed the consent sheet took part.

### Participants

As usual, during the Patient Safety module (MED5018), all final year medical students were randomly allocated to groups of 20 students. Each group was taught the IV fluid curriculum during an intensive 1-week teaching block.

All 240 students of the 2016-17 academic cohort were invited to take part in the study. The 178 students who accepted this invitation and signed informed consent forms were representative of the usual medical student cohort in terms of age and gender. Participating students were randomly allocated to either the intervention group (n=83) or the control group (n=95).

### Research instruments

A Multiple-Choice Questionnaire (MCQ) was created comprising 50 randomly selected questions from the University’s bank of examination questions related to the IV fluid curriculum. The MCQ was compiled in a PowerPoint presentation, using one slide per question. Paper copy answer sheets were designed for individual student completion.

**Figure 1.**
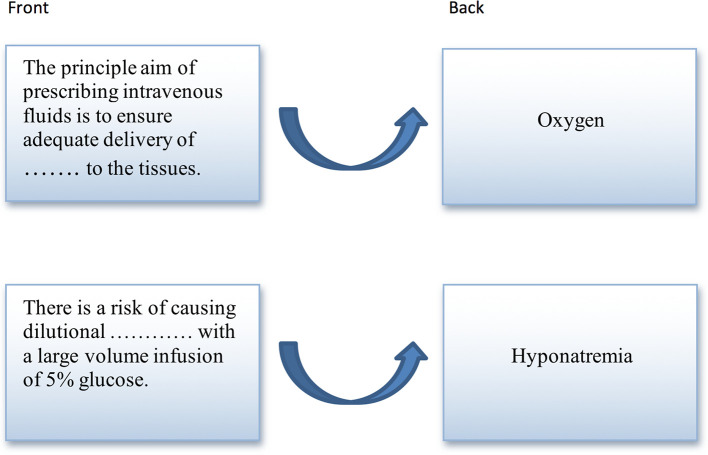
Example of 2 SAFMEDS cards, showing front and back sides

Laminated SAFMEDS (each pack containing 50 cards) were prepared for each participant in the intervention group. Each card contained a fill-in statement related to IV fluid therapy (e.g. fluid compartments, osmolality), with a key term or phrase missing. The reverse side of each card displayed the correct missing term/phrase ( Figure 1). Data collection sheets were printed out for students to record their SAFMEDS results.

### Procedure

During the 5-day teaching week (Monday to Friday), standard teaching methods were used for all students. These involved didactic lectures with interactive discussion delivered by the same lecturer (1 ^st^author) in all classes.

At baseline (i.e., Monday morning, prior to teaching), all students (intervention and control groups) completed the MCQ. The MCQ was displayed overhead for all students to see the same slide at the same time and for the same duration. Each student was given a paper copy response sheet to complete individually, without conferring with other students. All participants were assigned a unique identity number to ensure anonymity. At the end of the MCQ, completed response sheets were collected by the lecturer. Data were entered into Excel spreadsheet format for analysis.

Following the MCQ, the intervention group were given a brief information talk about the rationale and evidence of PT before they were instructed on how to use SAFMEDS. They were told that the objective was to work through as many SAFMEDS flashcards as possible within 1-minute trials and they were asked to repeat this process up to five times per day during the teaching week (Monday-Friday). A sufficient number of SAFMED packs (each containing 50 cards) was made freely available to all intervention group students for revision and study purposes.

Students completed up to five 1-minute trials per day. They set an alarm for 1 minute, read out the phrase on the front of each card andvocalisedthe missing term to complete the statement. They then quickly checked the back of each card to verify whether their response was correct or incorrect and placed the card on corresponding pile on the table in from of them; one pile for correct responses and one pile for incorrect responses. At the end of the minute, when the buzzer went off and the trial was finished, students counted the cards on each pile in front of them and recorded their results on their dated data sheet.

At the end of the week, on Friday afternoon, after the IV fluid curriculum teaching had been completed, both the control and intervention groups were re-tested using the same MCQ. In this post-test, conditions were held the same as during the pre-test on the first day (Monday), although the questions were shuffled.

To ensure equity across the entire student cohort after study completion, all students (i.e., those in control group and those who had not consented to participate in the study) were briefed on how to use SAFMEDS and access to SAFMEDS flashcards was made available to all students for personal study.

### Data analysis

Data were analysed using descriptive statistics (average percentage of correct and incorrect responses on MCQ for control versus intervention groups, individual student scores on MCQ expressed as percentage of correct responses pre and post-intervention) and graphs were presented for visual analysis ( What Works Clearing House, 2010).

## Results/Analysis

**Figure 2.**
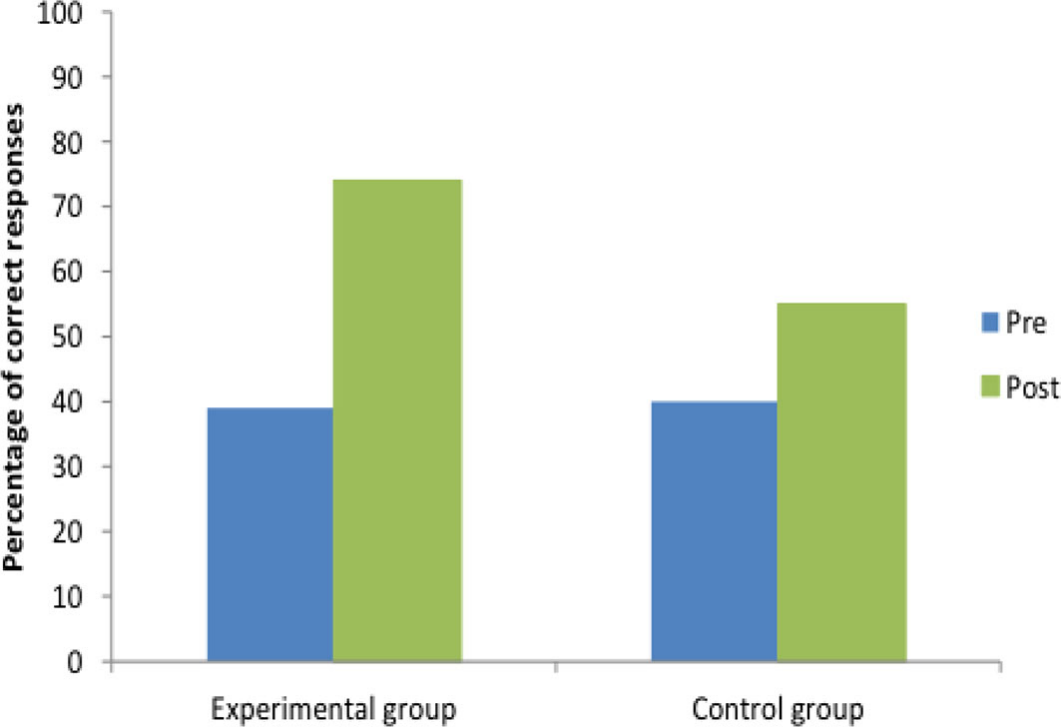
Percentage of correct and incorrect MCQ responses for control group and intervention group pre- and post-training

At baseline, MCQ accuracy scores for the intervention group and the control group were comparable, 39% and 40%, respectively. At post-intervention, there was a difference of 20 percentage points favouring the performance of the intervention group ( Figure 2). Specifically, the intervention group achieved a 35% improvement in accurate responding (increasing fluent responding from 39% to 74%), while the control group achieved a 15% improvement (increasing fluent responding from 40% to 55%). Inaccurate responses to the post-intervention MCQ decreased from 61% to 26% for the intervention group and from 60% to 45% for the control group.

**Figure 3.**
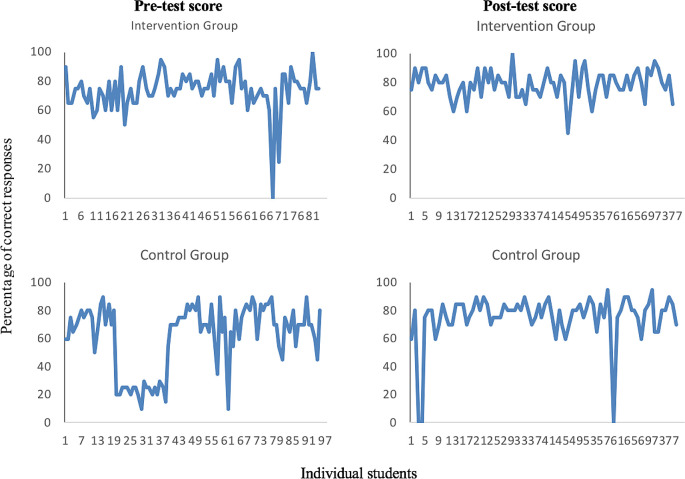
Percentage of correct MCQ responses at pre- and post-test for study group and control group

When comparing individual student scores in the MCQ pre- and post-intervention ( Figure 3), there was evidence that pre-intervention both groups had a number of failing students (UK pass mark at undergraduate level is 40%). Post-intervention, all students in the intervention group passed, with only one student scoring as low as 45% and all remaining students scoring between 60% and 100%. In the control group, three students failed the post-intervention MCQ scoring only between 10% and 35%, while the remaining control group students passed with grades ranging between 50% and 90%.

**Figure 4.**
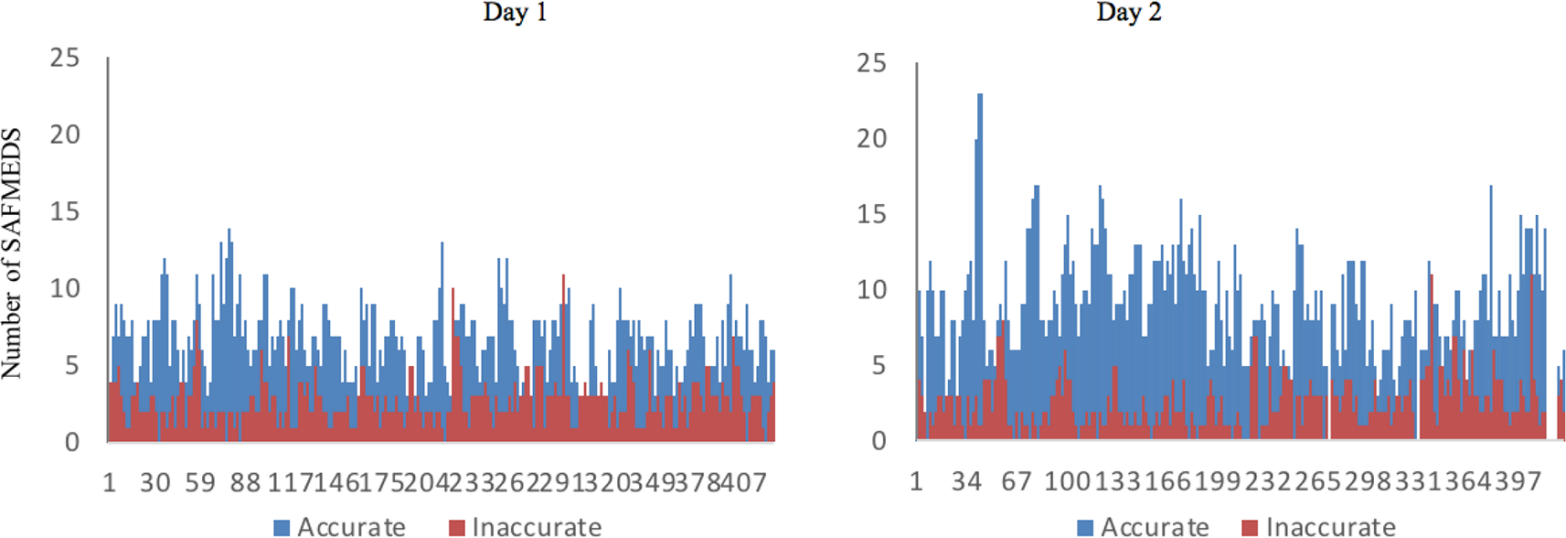
Accuracy and speed of SADMEDS cards completed during individual trails

During the intervention, students in the intervention group carried out 3-5 daily trials per day, Monday to Friday. The overall accuracy of completed SAFMEDS cards increased from 71.8% to 79.4%, with corresponding decreases of inaccurately answered SAFMEDS flashcards ( Figure 4). With regards to speed, data show marked increases in the number of cards completed per trial across trials for all students. The performance on the SAFMEDS in the intervention group improved from an average of 8 cards per trial to an average of 10 cards.

**Figure 5.**
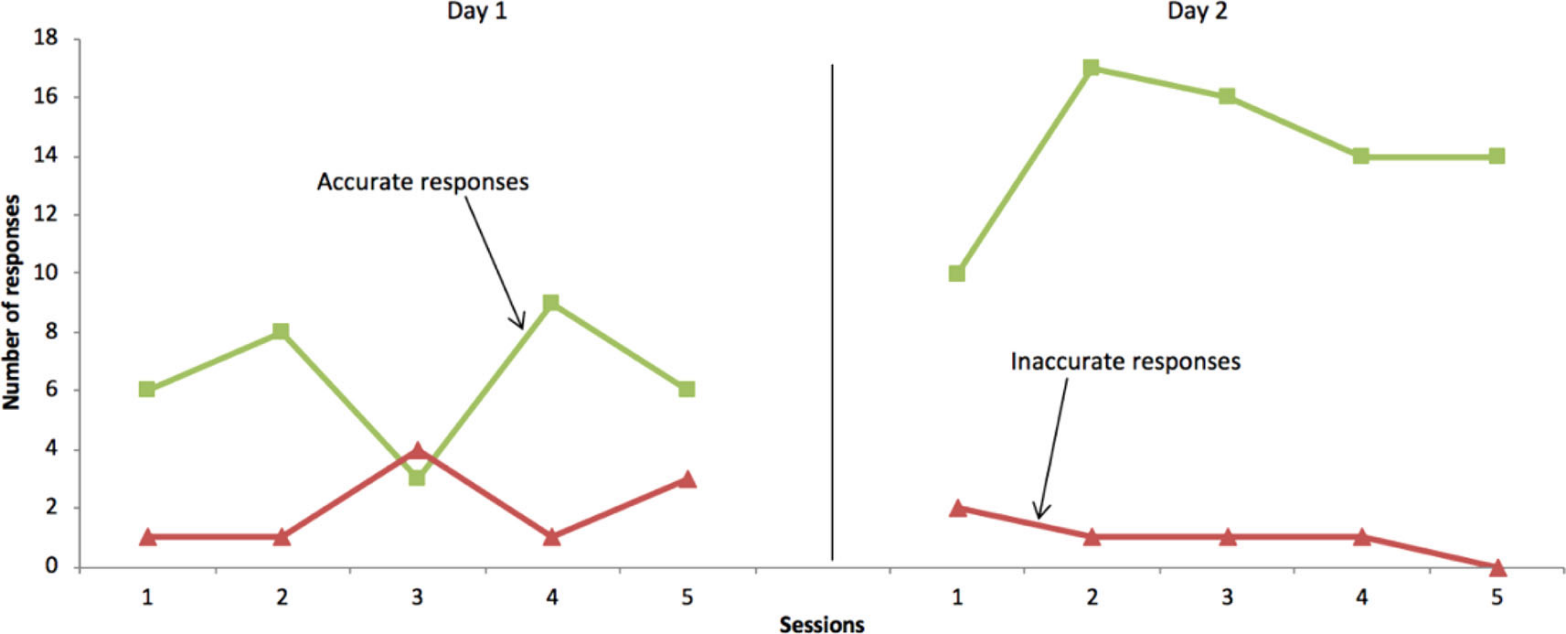
Frequency of accurate and inaccurate SAFMEDS responses for Student 23

To illustrate improvements during SAFMEDS trials, Figure 5 shows data for Student 23 across 5 daily trials for Monday and Wednesday during the intervention week. On the first teaching day (Monday) this student completed an average of 8 SAFMEDS per trial, with an average of 6 accurate and 2 inaccurate responses. On Wednesday, after further study, this student completed an average of 15 SAFMEDS per trial, with an average of 14 accurate and 1 inaccurate response.

## Discussion

Intravenous fluid therapy is a common medical inpatient intervention in hospitals and is usually the responsibility of relatively junior FY1 doctors. Given the importance of this procedure and the potentially serious consequence of medical errors in this area, effective training for medical students is crucial. The current study evaluated the effectiveness of a brief intervention based on the scientific discipline of applied behaviour analysis. Precision Teaching (PT), and more specifically SAFMEDS, was used to enhance fluency, i.e., accuracy and speed, of final year medical students’ performance related to IV fluid therapy. PT proved to be an effective adjunct to standard teaching in improving students’ knowledge in the area of IV fluids. Post-intervention, knowledge of IV fluid therapy was 20 percentage points higher in the intervention group when compared to the control group. The results showed a significant increase in accuracy and reduction in inaccurate responses when using SAFMEDS. Speed of completion of SAFMEDS increased markedly during the training week. The use of PT appeared particularly effective with weaker students, some of whom probably would have failed the MCQ without the additional training.

Precision Teaching is a relatively novel concept within medical education ( Lydon et al., 2017; McGrath et al., 2018), although its success is well documented in other areas of education and teaching ( Kubina, Morrison and Lee, 2002; Beverley, Hughes and Hastings, 2009). The present study adds weight to the existing evidence of the benefits using of SAFMEDS to teach knowledge content. Clearly, using PT in medical education has the potential to increase IV fluid therapy knowledge in junior doctors and thereby should lead to higher levels of patient safety.

As in all research, the present study has some limitations. While pre- and post-assessments and usual teaching were conducted by the same clinical lecturer (1st author), students self-monitored their performance while using SAFMEDS (i.e., they counted and recorded their own correct and incorrect responses). This may have introduced some inaccuracies in the data. However, given that each student conducted 3-5 SAFMEDS trials per day during up to 5 days during the intervention week, data were available on hundreds of trials. Therefore, it is unlikely that random errors would have led to significantly different overall results.

At the same time, the present study was conducted with one cohort of medical students at one specific UK Russell Group university. Given that medical education has a very long and successful history at this institution, it is to be expected that existing teaching methods were sophisticated and effective. Therefore, data reported here may underestimate the potential of PT methods when introduced in less well-established medical schools.

There are, of course, a number of issues that require further investigation. As the intervention was purely based on SAFMEDS flashcards, rather than on practical demonstrations of skills and the MCQ assessment was entirely paper-based, it would be interesting to see if the intervention would have a positive impact on student performance during Objective Structured Clinical Examination (OSCE) style exams. Such testing would shed light on the effect of PT- methods on practical skills and mastery in IV fluid therapy and it may show if the additional input would enable students to better interpret clinical findings and prescribe appropriately.

Undeniably, it would be important to explore also the long-term impact of the intervention on the retention of new knowledge, concepts, and skills. If fluency improvement can be maintained across time, patient characteristics, and location, it would be useful to trial a technology-based system to present SAFMEDS, such as a bespoke mobile application, whereby individual and group performance could be tracked efficiently, eliminating potential inaccuracies of self-reporting.

Having said this, the significant improvements in MCQ scores achieved by the intervention group together with the improvement in fluency during practice with SAFMEDS are very encouraging. Behaviour analytic teaching methods, such as PT, undoubtedly merit further exploration in other areas of higher education.

## Conclusion

Precision Teaching methods, specifically SAFMEDS, were used as an adjunct to standardized teaching to improve fluency, i.e., accuracy and speed, as well as overall performance of senior undergraduate medical students with regards to IV fluid therapy. The study provides support to the growing body of evidence in favour of the use of PT to achieve mastery and fluency. Our study showed that use of SAFMEDS in a relatively low number of speed trials can enhance the learning of medical students, regardless of their initial knowledge level. As such, the study adds to the evidence that applied behaviour analytic procedures offer promise in medical education.

## Take Home Messages

• The study provides support to the growing body of evidence in favour of the use of PT to achieve mastery and fluency.
• The use of SAFMEDS in a relatively low number of speed trials can enhance the learning of medical students.
• The study adds to the evidence that applied behaviour analytic procedures offer promise in medical education.

## Notes On Contributors

Ian K Walsh

Ian K Walsh, MD MSc FRCSUrol SFHEASchool of Medicine, Dentistry and Biomedical Sciences, Queen’s University Belfast. Mr Walsh is a Clinical academic at Queen’s University, Belfast. He is lead for the Healthcare Human Factors Group and the Transatlantic Arts, Humanities and Healthcare Network, with specialised clinical practices in Urology, Psychodynamics and Sexual Function.

Katerina Dounavi

Dr Katerina Dounavi is a Psychologist and Board Certified Behavior Analyst-Doctoral (BCBA-D®). She serves as a Lecturer in Behaviour Analysis and Autism and MScABA Director at Queen’s University Belfast. She has extensive experience in supervising behavioural interventions and conducting staff training internationally. Her research interests focus on applied behaviour analysis, developmental delays including autism, evidence-based education, inclusion, and behavioural interventions targeting health and wellbeing.

**Joseph Houghton**

Dr Joseph Houghton, MB BCh BAO FRCPath BSc Medical Genetics MSc Clinical Education, is a Clinical Senior Lecturer in the Centre for Medical Education, School of Medicine, Dentistry and Biomedical Sciences Queen’s University Belfast. He is the academic lead for pathology in the undergraduate medical curriculum and is an external examiner for the Royal College of Pathologists, London.

Kathy M Cullen

Dr Kathy Cullen is a Clinical Senior Lecturer (Education) in the Centre for Medical Education at Queen’s University Belfast and a Consultant Respiratory Physician in the Belfast Trust. She is Lead for Year 3 assessment and Deputy Lead for Final year clinical exams and directs two major near-peer teaching programmes in the medical school.

Karola Dillenburger

Prof. Karola Dillenburger is Professor of Behaviour Analysis and Education and Director of the Centre for Behaviour Analysis at Queens University Belfast. She is a Board Certified Behaviour Analyst-Doctoral and Clinical Psychologist (Health Care Professional Council registered). She is the Course Director of the MSc in Autism Spectrum Disorders (ASD) and a member of the Northern Ireland Autism Strategy Regional Multi-Agency Implementation Team (ASRMAIT). ORCID: https://orcid.org/0000-0002-3410-5949

## Declarations

The author has declared that there are no conflicts of interest.

## Ethics Statement

Ethical approval was granted from the School of Medicine, Queen’s University Belfast - Reference: 14.10v2. The study was conducted in adherence to QUB Research Governance Guidelines and all relevant data protection procedures were implemented. Only students who had signed the consent sheet took part.

## External Funding

This article has not had any External Funding
