# Supplementary material for: Fluency training in medical education: Improving competence in IV fluid therapy knowledge and skills
Source: MedEdPublish (2016). 2019 Jan 29;8:23. [Version 1] doi: 10.15694/mep.2019.000023.1 (PMC10712452; doi:10.15694/mep.2019.000023.1)
Supplement: Peer review history

# Reviewer report: Reviewer response for version 1

**DOI:** 10.21956/mep.19758.r29659

**Published:** 2019-02-10

**Authors:** Madalena Folque Patricio

**Affiliations:** 1 School of Medicine, University of Lisbon

This review has been migrated. The reviewer awarded 4 stars out of 5 Thanks for the opportunity of reading this interesting study. As it happened with a co-reviewer, this is my first introduction to Precision Teaching and to the SAFMEDS flash cards. The study is well designed and clearly reported, but improvements would be welcome namely in what concerns data analysis and presentation of results. For data analysis (as already suggested by another co-reviewer), a t-test should have been performed to check the significant difference between the groups with indication of effect size. The difference in pre and post clearly percentages indicates that there were improvements but the t-test is needed. Without it the authors should not say that 'the significant improvements in MCQ scores achieved by the intervention group together with ...etc.' Regarding the presentation of results, Figures 3 and 4 should be improved, as they are unclear. In Fig 3, what means the ‘0’ values? Does ‘0’ mean that students failed all questions (which is not probable) or that students missed the pre or post-test? Why are the ‘points' representing students’ performance linked? If the authors decide to keep this figure, it would be better to have students represented by 'points' not linked and presented by increasing values to facilitate the comparison of groups. I propose a Scatter Plot to be presented. Fig. 4 is also unclear and in terms of Fig.5 what is the objective underlying the presentation of results for just one student (student 23)? Why and how was ‘student 23’ selected? There are other areas for improvement but I was pleased to see that some were identified by the authors and reported under ‘study limitations’: the first one concerns testing students at a later stage for follow-up results (for example 3 or 6 months later) while the second is to assess other domains rather than just knowledge (for example using an OSCE, other practical exam, etc.). To conclude, let me recommend this interesting paper to readers and thank the authors for the opportunity to learn more in medical education.

Reviewer Expertise:

NA

No decision status is available

# Reviewer report: Reviewer response for version 1

**DOI:** 10.21956/mep.19758.r29657

**Published:** 2019-02-01

**Authors:** Trevor Gibbs

**Affiliations:** 1 AMEE

This review has been migrated. The reviewer awarded 3 stars out of 5 I agree with the comments from my co-reviewers- this is an interesting paper about a subject that is not frequently taught in the best way and as a complicated subject, one that maybe not very well learned. I think we have all been in the situation when we learn more as a postgraduate when we are faced with the problem instead of learning from the teaching. Any method that can improve the learning of IV therapy is and should be welcomed.I think this is the second paper recently published in MedEdPublish that used the SAFMEDS falsh cards. My worry lies in the low level of evaluation and I would agree that a more longitudinal approach to evaluation would give me more confidence in the methodology.

Reviewer Expertise:

NA

No decision status is available

# Reviewer report: Reviewer response for version 1

**DOI:** 10.21956/mep.19758.r29658

**Published:** 2019-02-01

**Authors:** Megan Anakin

**Affiliations:** 1 University of Otago

This review has been migrated. The reviewer awarded 3 stars out of 5 Thank you for inviting me to review this article. Teachers will be interest in reading about the teaching method described in this study. The authors clearly outline the need to improve student learning about IV fluid therapy. The title signposts that the study is about improving competence in IV fluid therapy knowledge and skills, however, the teaching intervention focuses on factual recall of knowledge rather than the performance of skills. Even though, the intervention was brief, only one week in duration, the pre- and post-testing results were interpreted to indicate that the intervention had an impact on student performance. Missing from the analysis of the data was the use of inferential statistics, (e.g., reporting the results of a t-test and calculation of an effect size) to report if there was a statistically significant difference in achievement between the control and intervention groups and the magnitude of that difference. Box and whisker plots would also help to report the data shown in Figure 2.To enhance the discussion section, the authors could relate their results to the “growing body of evidence in favour of the use of PT to achieve mastery and fluency” mentioned in the conclusion. I’m wondering what the results might be like if participants were tested again 6 weeks or 3 months later. I’m also wondering how students’ knowledge IV fluid therapy might be applied to improve their clinical practice. Thank you publishing this thought-provoking article.

Reviewer Expertise:

NA

No decision status is available

# Reviewer report: Reviewer response for version 1

**DOI:** 10.21956/mep.19758.r29660

**Published:** 2019-01-30

**Authors:** P Ravi Shankar

**Affiliations:** 1 American International Medical University

This review has been migrated. The reviewer awarded 4 stars out of 5 This is an interesting study about the use of precision teaching (PT) to improve competence in IV fluid therapy knowledge and skills among final year undergraduate medical students. The methodology has been well described and can be replicated elsewhere. This was my first introduction to precision teaching and to the SAFMEDS flash cards. The strengths and limitations of the study have been well described. The methods shows promise to teach IV fluid therapy and other topics in medicine. The study will be of interest to all medical educators.

Reviewer Expertise:

NA

No decision status is available
